# Comparison of postoperative analgesic effects of ultrasound-guided intercostal nerve block and transversus abdominis plane block in patients undergoing laparoscopic cholecystectomy: randomized clinical trial

**DOI:** 10.1093/bjsopen/zraf022

**Published:** 2025-07-01

**Authors:** Hongchun Xu, Dandan Song, Zhiqiang Wu, Chao Lin, Wuchang Fu, Fangjun Wang

**Affiliations:** Department of Anaesthesiology, Affiliated Hospital, North Sichuan Medical College, Nanchong, China; Department of Anaesthesiology, Affiliated Hospital, North Sichuan Medical College, Nanchong, China; Department of Anaesthesiology, North Sichuan Medical College, Nanchong, China; Department of Anaesthesiology, North Sichuan Medical College, Nanchong, China; Department of Anaesthesiology, Second Clinical Medical Institution of North Sichuan Medical College (Nanchong Central Hospital), Nanchong, China; Department of Anaesthesiology, Affiliated Hospital, North Sichuan Medical College, Nanchong, China

## Abstract

**Background:**

The aim of this study was to compare the postoperative analgesic effects of ultrasound-guided intercostal nerve block and transversus abdominis plane block in patients undergoing laparoscopic cholecystectomy.

**Methods:**

Patients undergoing laparoscopic cholecystectomy for chronic cholecystitis with gallstones were randomly allocated to ultrasound-guided T7–11 intercostal nerve block or subcostal transversus abdominis plane block (both with 40 ml 0.3% ropivacaine). The primary outcome was the dose of tramadol required for remedial analgesia 24 h after surgery. The secondary outcomes included visual analogue scale scores at different time points after surgery, the time of initial use of tramadol for postoperative analgesia, patient satisfaction with postoperative pain control, the time to flatus, and the incidence of postoperative adverse events.

**Results:**

A total of 64 patients were included. Compared with the transversus abdominis plane block group, the intercostal nerve block group had lower visual analogue scale scores at 3 h after surgery (mean(s.d.) of 2.4(0.8) *versus* 1.6(0.6)), 6 h after surgery (mean(s.d.) of 2.2(0.3) *versus* 1.4(0.6)), and 8 h after surgery (mean of 1.7(0.5) *versus* 1.3(0.4)) (*P* < 0.001, *P* < 0.001, and *P* = 0.002 respectively), a lower dose of tramadol for remedial analgesia within 24 h after surgery (median of 100 (interquartile range 0–100) *versus* 50 (interquartile range 0–50) mg) (*P* = 0.012), and a significantly delayed time of initial use of tramadol for postoperative analgesia (mean(s.d.) of 9.1(7.5) *versus* 14.6(8.3) h) (*P* = 0.015). The incidences of postoperative dizziness and postoperative nausea and vomiting were higher in the transversus abdominis plane block group (47% and 69% respectively) than in the intercostal nerve block group (19% and 41% respectively) (*P* = 0.032 and 0.035 respectively). Patient satisfaction with postoperative analgesia was higher in the intercostal nerve block group than in the transversus abdominis plane block group (*P* = 0.037). The time to flatus was similar between the two groups (*P* > 0.050).

**Conclusion:**

Compared with ultrasound-guided subcostal transversus abdominis plane block, ultrasound-guided T7–11 intercostal nerve block with 0.3% ropivacaine provides better postoperative analgesia, requires a lower dose of tramadol for remedial analgesia 24 h after surgery, and significantly delays the time of initial use of tramadol for postoperative analgesia.

## Teaser

Moderate to severe postoperative acute pain occurs in 80% of patients undergoing laparoscopic cholecystectomy due to intraoperative trauma and pneumoperitoneum. Appropriate postoperative analgesia is necessary for patients undergoing laparoscopic cholecystectomy. Compared with subcostal transversus abdominis plane block, ultrasound-guided T7–11 intercostal nerve block with 0.3% ropivacaine provides better postoperative analgesia, requiring a lower dose of tramadol for remedial analgesia 24 h after surgery.

## Introduction

Laparoscopic cholecystectomy (LC) is a minimally invasive procedure with a short surgical time and a rapid postoperative recovery. In recent years, LC has gradually become a day-case surgery, with the development of postoperative enhanced recovery. Sufficient analgesia and enhanced recovery are the primary reasons for the increasing rate of day-case surgery^1^. Moderate to severe postoperative acute pain occurs in 80% of patients undergoing LC due to intraoperative trauma and pneumoperitoneum^2^. Postoperative pain may result in infection, cognitive impairment, prolonged wound healing, prolonged hospital stay, and even chronic postoperative pain^3^. Therefore, appropriate postoperative analgesia is necessary for patients undergoing LC.

Transversus abdominis plane (TAP) block, as part of postoperative multimodal analgesia, has been widely used in clinical practice. TAP block induces sensory block by targeting the spinal nerve fibres with local anaesthetics in the plane between the internal oblique and transversus abdominis muscles. The anterior branch of the thoracic spinal nerve constitutes the intercostal nerve and intercostal nerves T7–12 are segmentally distributed to the abdominal skin. Blocking of the right T7–11 intercostal nerve at the lower edge of the ribs laterally in the midaxillary line leads to muscle relaxation and loss of skin sensation in the corresponding segment of the surgical site^4^. Therefore, both TAP block and T7–11 intercostal nerve block (ICNB) seem to provide postoperative analgesia in patients undergoing LC.

However, with regard to TAP block, the interfascial plane is uneven and the block effect is influenced by muscle tension, posture, and respiratory movement^5^. Due to the fact that the intercostal nerves run through the rib groove, which is located at the lower edge of the ribs, it is easier to accurately locate the intercostal nerves during ICNB and the block effect is more precise. No reports have compared the two postoperative analgesia methods for LC. The aim of this study was to compare the postoperative analgesic effects of TAP block and T7–11 ICNB in patients undergoing LC.

## Methods

### Study overview

This clinical trial was approved by the Ethics Committee of Nanchong Central Hospital (Approval No. 2022.37) and written informed consent was obtained from all study participants. This prospective, double-blind, randomized, controlled study was registered before patient enrolment in the Chinese Clinical Trial Registry (https://www.chictr.org.cn/showproj.html?proj=170842; registration number ChiCTR2200060302; principal investigator W.F.; date of registration 26 May 2022). This clinical study adhered to the CONSORT guidelines^6^.

### Enrolment

From July 2022 to May 2023, the first patient that underwent LC every day in Nanchong Central Hospital was screened for inclusion in the study. Patients diagnosed with chronic cholecystitis with gallstones and undergoing elective LC were included. Further inclusion criteria were: ASA grade I or II; aged 18–65 years; and a BMI of 18–30 kg/m^2^. The exclusion criteria were as follows: coagulation disorders; a history of allergy to ropivacaine and lidocaine; infection or tumour at the puncture site; a history of trauma or long-term low back pain; neuropsychiatric disorders; thoracic deformity; a history of drug or alcohol addiction; participants who declined to sign informed consent forms; communication difficulties; recent acute attacks of cholecystitis or concomitant ventral hernias; and pregnant, breastfeeding, or menstruating women. The withdrawal criteria were as follows: changes in surgical procedures, postoperative loss to follow-up; incomplete data collection.

### Randomization and blinding

Randomization was performed by an anaesthesia assistant who was not involved in the study, using a random number table. A total of 64 two-digit numbers, randomly selected from a random number table, were arranged in order from small to large. The anaesthesiologist set serial numbers of 1–32 in the TAP block group and 33–64 in the ICNB group. Patient grouping information was written on a card, which was sealed in an opaque envelope. Outcomes were assessed by a trained anaesthetic nurse who was blinded to the treatment allocation. Surgeons, anaesthesiologists, and other ward staff members were blinded to the allocation.

### Analgesic and anaesthetic techniques

Patients were assessed before surgery using laboratory evaluations (complete blood picture, renal function tests, and liver function), physical examination, and medical history, and were interviewed the day before surgery. The research protocol was explained to each participant, including the TAP block and ICNB procedures. All patients were made familiar with the use of the numeric rating scale for satisfaction (NRSS), with one corresponding to very dissatisfied and four corresponding to very satisfied^7^, and the visual analogue scale (VAS), which is assessed on a 10 cm horizontal line, with the right-hand end representing the most severe pain imaginable and the left-hand end representing no pain^8^. All patients fasted before surgery for 8 h for solid food and 6 h for clear liquids and had no pre-anaesthetic medication.

Monitoring in the operating room included non-invasive blood pressure (NIBP), ECG, peripheral oxygen saturation (SpO_2_), urine volume, heart rate (HR), and respiratory rate. After upper-extremity intravenous access was secured, all patients received an intraoperative infusion of 8–10 ml/kg Ringer’s lactate solution per hour. Anaesthesia was induced using intravenous fentanyl (4 μg/kg), propofol (2 mg/kg), and cisatracurium (0.15 mg/kg). After tracheal intubation, controlled mechanical ventilation was precisely adjusted to maintain end-tidal CO_2_ concentrations of 35–45 mmHg. Anaesthesia was maintained during surgery with a remifentanil plasma target concentration of 3–6 ng/ml and a propofol plasma target concentration of 1.5–4 μg/ml according to a bispectral index value of 40–60 in the two groups.

**Fig. 1:**
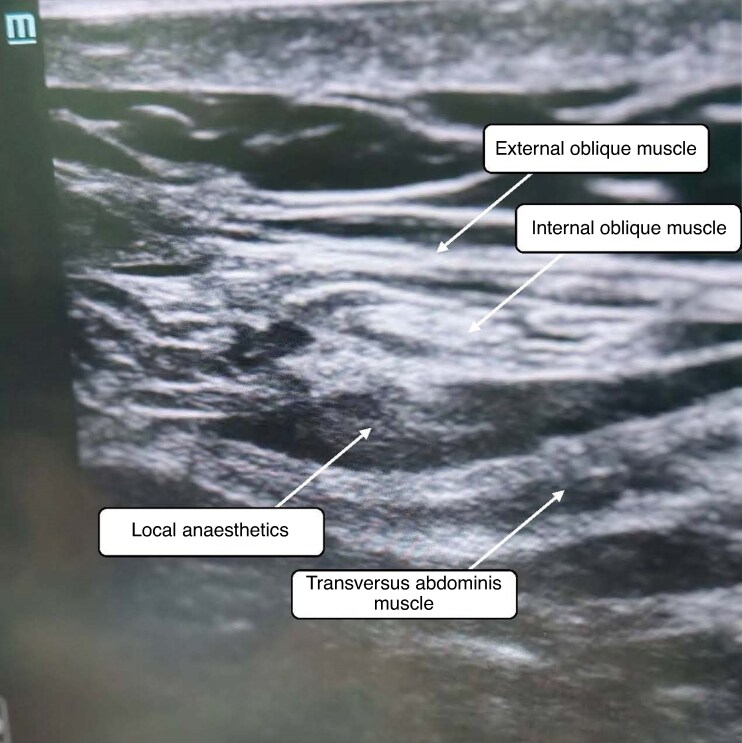
Ultrasound-guided transversus abdominis plane block

After induction of anaesthesia, ultrasound-guided TAP block was performed using an oblique subcostal approach in the TAP block group. A linear ultrasonography probe was placed on the upper abdominal wall near the xiphisternum of the sternum and moved laterally along the subcostal margin until the obliquus externus abdominis, obliquus internus abdominis, transversus abdominis, and fascia were identified^9^. The anaesthesiologist directed the needle with an in-plane technique toward the transversus abdominis fascia and 20 ml 0.3% ropivacaine was injected between the transversus abdominis and rectus abdominis muscles within 30 s, as shown in *Fig. 1*. The same procedure was performed using the same concentration and volume of ropivacaine solution on the contralateral side.

**Fig. 2:**
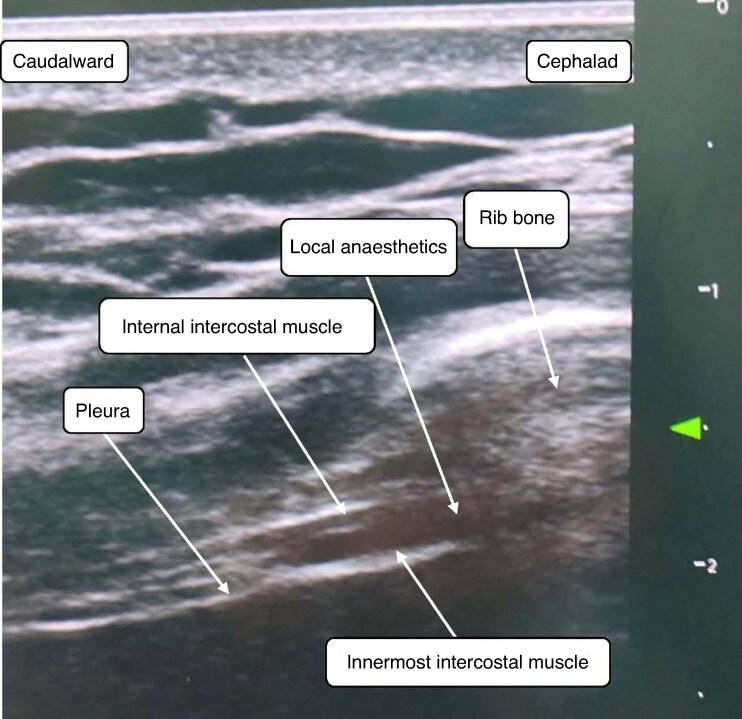
Ultrasound-guided intercostal nerve block

After induction of anaesthesia, ultrasound-guided ICNB was performed in the ICNB group^10^. An ultrasonography probe was placed on the axillary midline of the lateral thoracic wall at an angle of 30–45° to the midaxillary line and the ribs and pleura were identified, as shown in *Fig. 2*. A 22-G short-bevel needle was used under ultrasonography guidance and, when the needle tip arrived at the plane between the internal intercostal muscle and the innermost intercostal muscle, 5 ml 0.3% ropivacaine was injected into each intercostal bundle without withdrawing air or blood. This procedure was performed bilaterally between intercostal T7 and T11. Each patient received a total of eight injections.

All blocks were performed under aseptic technique by an anaesthesiologist who was skilled in these procedures.

### Surgical technique and postoperative care

Three-port LC was performed after the nerve blocking procedure was completed in both groups. CO_2_ with a pressure of 10–12 mmHg was used to establish pneumoperitoneum during surgery. The residual CO_2_ gas in the abdominal cavity was eliminated completely by pressure on the abdomen or suction with a negative pressure suction tube at the end of surgery. ICNB, TAP block, and surgery sites were covered after surgery for all patients in both groups. Consequently, the surgeon, anaesthetic nurse who assessed the outcomes, and ward staff did not know the group to which each patient belonged. Patients were transferred to the post-anaesthesia care unit (PACU) after surgery and discharged to the surgical ward when the Steward score was greater than four^11^. When the VAS score was greater than three and the patient requested analgesia in the PACU or surgical ward, tramadol (50 mg) was injected intravenously^12^. If pain was not relieved, another dose of tramadol (50 mg) was administered.

### Outcomes

The primary outcome was the postoperative dose of tramadol required for remedial analgesia. The secondary outcomes were systolic blood pressure (SBP), diastolic blood pressure (DBP), HR, and peripheral SpO_2_ at 5 min before surgery (T_0_), skin incision (T_1_), CO_2_ pneumoperitoneum (T_2_), during surgery (T_3_), end of surgery (T_4_), 3 h after surgery (T_5_), 6 h after surgery (T_6_), 8 h after surgery (T_7_), and 24 h after surgery (T_8_), the postoperative VAS scores at T_5_, T_6_, and T_7_, the first tramadol administration time, the time to flatus after surgery, the satisfaction with postoperative analgesia (assessed using a four-point Likert scale: 1, very dissatisfied; 2, somewhat dissatisfied; 3, somewhat satisfied; and 4, very satisfied)^13^, and the incidence of postoperative adverse events.

### Sample size calculation

No studies on postoperative analgesia with ICNB after LC and remedial analgesia with tramadol were available; therefore, the sample size calculation in this study was based on a pilot study of ten patients who underwent LC in each group. The mean(s.d.) postoperative doses of tramadol for remedial analgesia in the TAP block and ICNB groups were 75(42.5) and 40(31.6) mg respectively. Assuming a two-sided type I error of 0.050 and a type II error of 0.100, which eventually results in a power of 0.90 (1 − β), the sample size of each group was calculated to be 26 using PASS 15.0 software. Considering a dropout rate of 20%, 33 patients were required in each group.

### Statistical analysis

Statistical analyses were conducted using SPSS^®^ (IBM, Armonk, NY, USA; version 23.0). Quantitative variables with a normal distribution are presented as mean(s.d.), quantitative variables with a non-normal distribution are presented as median (interquartile range (i.q.r.)), and enumeration data are presented as *n* (%). The clinical characteristics and demographic data of the two groups were compared using the Mann–Whitney *U* test, the chi-squared test, and Student’s *t* test as appropriate. Repeated measures ANOVA was used for VAS scores, SBP, DBP, HR, and SpO_2_ to observe the potential variation over time and post-hoc analysis was performed to detect pairwise differences using Bonferroni correction. *P* < 0.050 was considered statistically significant.

## Results

**Fig. 3:**
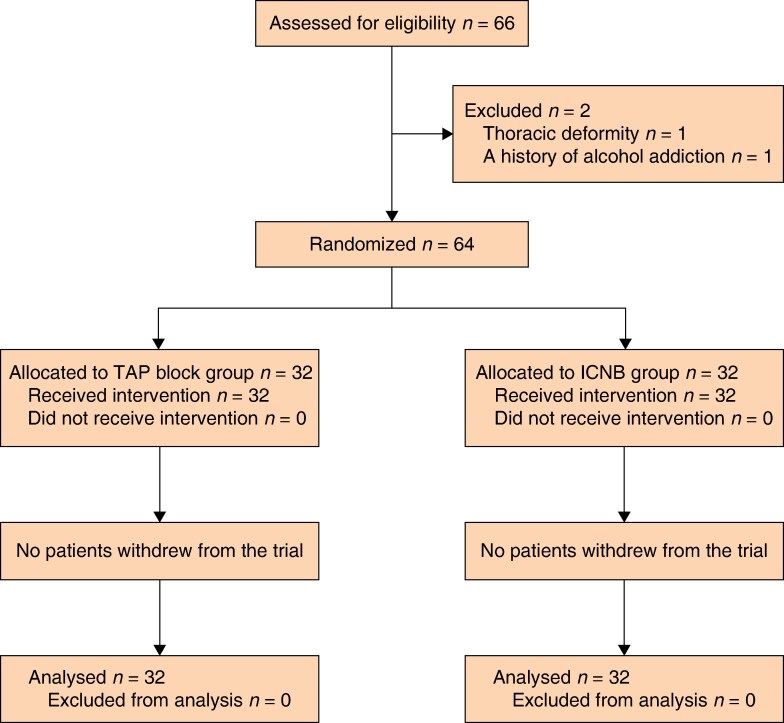
Participant flow diagram

During the study interval, 685 patients underwent LC and a total of 66 patients were screened for eligibility; one patient with thoracic deformity and one patient with a history of alcohol addiction were excluded and thus 64 patients were allocated to the two groups and completed the study (*Fig. 3*). The demographic profiles of the patients in the two groups are shown in *Table 1*. The demographic profiles of the patients in the two groups were similar (*P* > 0.050).

**Table 1:** Demographic data

	TAP block group (*n* = 32)	ICNB group (*n* = 32)	*F*/Χ^2^	*P*
Age (years), mean(s.d.)	39.8(7.3)	38.6(8.9)	0.246	0.622
Weight (kg), mean(s.d.)	58.5(5.8)	59.7(6.1)	0.454	0.503
**Sex**				
Male	13 (41)	15 (47)	0.254	0.614
Female	19 (59)	17 (53)		
**ASA grade**				
I	24 (75)	22 (69)	0.309	0.578
II	8 (25)	10 (31)		
Height (cm), mean(s.d.)	163.3(5.3)	162.3(5.0)	0.355	0.554
Duration of anaesthesia (min), mean(s.d.)	69.2(7.2)	67.9(8.8)	0.258	0.613
Duration of PACU (min), mean(s.d.)	18.8(2.6)	18.3(2.3)	0.293	0.590
Duration of surgery (min), mean(s.d.)	49.9(6.1)	48.5(7.2)	0.432	0.513
Duration of hospital stay (days), mean(s.d.)	2.6(0.7)	2.4(0.6)	0.459	0.500

Values are *n* (%) unless otherwise indicated. TAP, transversus abdominis plane; ICNB, intercostal nerve block; PACU, post-anaesthesia care unit.

The postoperative dose of tramadol was significantly lower in the ICNB group (median of 50 (i.q.r. 0–50)) than in the TAP block group (median of 100 (i.q.r. 0–100)) (*P* < 0.050). Compared with the TAP block group, the first tramadol administration time was significantly delayed in the ICNB group (mean(s.d.) of 9.1(7.5) h in the TAP block group *versus* 14.6(8.3) h in the ICNB group) (*P* < 0.050). The proportion of patients requiring postoperative tramadol analgesia was higher in the TAP block group (75%) than in the ICNB group (56%), as shown in *Table 2*. The VAS scores in the two groups decreased gradually from T_5_ to T_8_ (*P* < 0.050). VAS scores at T_5_ to T_7_ were significantly lower in the ICNB group than in the TAP block group (*P* < 0.050), as shown in *Table 3*.

**Table 2:** Postoperative analgesia requirements within 24 h

	TAP block group (*n* = 32)	ICNB group (*n* = 32)	*F*/Χ^2^	*P*
Dose of tramadol (mg), median (i.q.r.)	100 (0–100)	50 (0–50)*	2.524	0.012
First tramadol administration time (h), mean(s.d.)	9.1(7.5)	14.6(8.3)*	6.209	0.015
**Postoperative use of tramadol (mg)**				
0	8 (25)	14 (44)	0.309	0.578
50	9 (28)	11 (34)		
100	11 (34)	7 (22)		
150	4 (13)	0 (0)		

Values are *n* (%) unless otherwise indicated. *P < 0.050 *versus* TAP block group. TAP, transversus abdominis plane; ICNB, intercostal nerve block; i.q.r., interquartile range.

**Table 3:** Visual analogue scale scores at 3–24 h after surgery

Time point	TAP block group (*n* = 32)	ICNB group (*n* = 32)	*F*	*P*
T_5_	2.4(0.8)	1.6(0.6)*	17.522	<0.001
T_6_	2.2(0.3)	1.4(0.6)*	31.843	<0.001
T_7_	1.7(0.5)	1.3(0.4)*	10.026	0.002
T_8_	1.1(0.4)	1.0(0.3)	0.472	0.495
*F*	17.392	7.321	–	–
*P*	<0.001	<0.001	–	–

Values are mean(s.d.) unless otherwise indicated. *P < 0.050 *versus* TAP block group. TAP, transversus abdominis plane; ICNB, intercostal nerve block; T_5_, 3 h after surgery; T_6_, 6 h after surgery; T_7_, 8 h after surgery; T_8_, 24 h after surgery.

There were no differences in SBP, DBP, HR, and SpO_2_ between the two groups at T_0_ to T_8_ (*P* > 0.050), as shown in *Table 4*. Satisfaction with postoperative analgesia was higher in the ICNB group than in the TAP block group (*P* < 0.050), as shown in *Table 5*. There was no difference in the time to flatus between the two groups (*P* > 0.050). The incidences of postoperative dizziness and postoperative nausea and vomiting were higher in the TAP block group (47% and 69% respectively) than in the ICNB group (19% and 41% respectively) (*P* < 0.050), as shown in *Table 6*. None of the patients in either group experienced local anaesthetic toxicity, respiratory depression, haematoma, or postoperative pruritus.

**Table 4:** Systolic blood pressure, diastolic blood pressure, heart rate, and oxygen saturation at 0–24 h

Time point	TAP block group	ICNB group	*F*	*P*
**SBP (mmHg)**				
T_0_	123.6(10.8)	122.9(10.1)	0.048	0.827
T_1_	116.1(9.4)	114.3(7.7)	0.502	0.481
T_2_	113.5(8.8)	116.2(8.2)	1.088	0.301
T_3_	115.8(9.2)	114.8(7.4)	0.184	0.669
T_4_	114.3(8.6)	113.3(6.9)	0.181	0.672
T_5_	122.4(9.2)	118.5(8.0)	2.165	0.146
T_6_	121.9(9.8)	118.4(7.7)	1.707	0.196
T_7_	121.6(9.7)	119.7(8.0)	0.509	0.478
T_8_	122.2(10.3)	120.4(8.6)	0.422	0.518
*F*	1.687	3.262	–	–
*P*	0.101	0.001	–	–
**DBP (mmHg)**				
T_0_	68.2(6.3)	67.4(7.1)	0.174	0.678
T_1_	64.7(6.6)	63.9(5.7)	0.192	0.663
T_2_	65.9(6.3)	66.0(6.5)	0.007	0.936
T_3_	67.0(6.9)	65.5(6.8)	0.539	0.466
T_4_	67.8(6.7)	66.0(6.7)	0.823	0.368
T_5_	65.6(6.5)	64.8(6.3)	0.149	0.701
T_6_	67.9(6.9)	66.8(6.7)	0.274	0.603
T_7_	68.6(7.0)	66.7(6.8)	0.803	0.374
T_8_	69.1(6.8)	67.0(6.9)	1.082	0.302
*F*	1.115	0.674	–	–
*P*	0.353	0.714	–	–
**HR (beats per minute)**				
T_0_	71.2(10.7)	72.1(9.6)	0.249	0.620
T_1_	72.4(11.1)	71.8(9.8)	0.401	0.529
T_2_	73.0(11.5)	72.6(10.0)	0.130	0.719
T_3_	72.8(11.3)	71.8(9.5)	0.144	0.706
T_4_	71.9(11.2)	71.5(9.7)	0.229	0.634
T_5_	72.4(11.4)	71.9(9.6)	0.164	0.687
T_6_	72.3(11.3)	72.0(9.8)	0.396	0.531
T_7_	71.1(11.0)	72.8(10.2)	0.276	0.601
T_8_	71.9(10.7)	72.2(10.3)	0.253	0.617
*F*	0.481	0.975	–	–
*P*	0.869	0.366	–	–
**SpO_2_ (%)**				
T_0_	97.9(0.4)	98.0(0.8)	0.059	0.809
T_1_	99.1(0.5)	99.3(0.6)	0.808	0.372
T_2_	98.9(0.6)	98.9(0.4)	0.031	0.860
T_3_	98.8(0.7)	98.1(0.3)	2.659	0.108
T_4_	98.7(0.6)	98.3(0.5)	2.426	0.124
T_5_	96.7(0.9)	97.2(0.8)	3.679	0.060
T_6_	97.0(0.7)	97.5(0.8)	3.940	0.052
T_7_	97.6(0.6)	97.8(0.7)	1.371	0.246
T_8_	97.7(0.7)	97.8(0.8)	0.232	0.631
*F*	32.780	15.764	–	–
*P*	0.00	0.00	–	–

Values are mean(s.d.) unless otherwise indicated. TAP, transversus abdominis plane; ICNB, intercostal nerve block; SBP, systolic blood pressure; T_0_, 5 min before surgery; T_1_, skin incision; T_2_, CO_2_ pneumoperitoneum; T_3_, during surgery; T_4_, end of surgery; T_5_, 3 h after surgery; T_6_, 6 h after surgery; T_7_, 8 h after surgery; T_8_, 24 h after surgery; DBP, diastolic blood pressure; HR, heart rate; SpO_2_, oxygen saturation.

**Table 5:** Satisfaction with postoperative analgesia

Four-point Likert scale	TAP block group (*n* = 32)	ICNB group (*n* = 32)	Χ^2^	*P*
Four (very satisfied)	10 (31)	18 (56)	7.835	0.037
Three (somewhat satisfied)	10 (31)	11 (34)		
Two (somewhat dissatisfied)	9 (28)	3 (10)		
One (very dissatisfied)	3(10)	0 (0)		

Values are *n* (%) unless otherwise indicated. TAP, transversus abdominis plane; ICNB, intercostal nerve block.

**Table 6:** Time to flatus and incidences of postoperative dizziness and postoperative nausea and vomiting

	TAP block group (*n* = 32)	ICNB group (*n* = 32)	*F*/Χ^2^	*P*
Time to flatus (h), mean(s.d.)	23.0(3.8)	22.7(4.1)	0.103	0.750
Dizziness	15 (47)	6 (19)*	5.741	0.032
**Nausea and vomiting**				
Without nausea or vomiting	10 (31)	19 (59)	6.704	0.035
Nausea	22 (69)	13 (41)		
Vomiting	12 (38)	6 (19)		

Values are *n* (%) unless otherwise indicated. *P < 0.050 *versus* TAP block group. TAP, transversus abdominis plane; ICNB, intercostal nerve block.

## Discussion

In this study, compared with the TAP block group, a significantly lower dose of tramadol is required for remedial analgesia 24 h after surgery and the first tramadol administration time is significantly delayed in the ICNB group. The postoperative VAS scores in both groups decrease gradually and the VAS scores in the ICNB group at 3, 6, and 8 h after surgery are significantly lower than those in the TAP block group. The incidences of postoperative dizziness and postoperative nausea and vomiting increase significantly in the TAP block group. However, the time to flatus is similar between the two groups.

LC is a common procedure and has become the procedure of choice for the surgical treatment of gallbladder disease^14^. Although LC is a minimally invasive surgery, it can cause moderate to severe postoperative pain. Postoperative pain includes incisional pain at the trocar site on the abdominal wall, diaphragmatic pain, local visceral pain, and referred visceral pain. Most of these nociceptive stimulations related to LC that cause postoperative pain are carried to the central nervous system through the T7–10 intercostal nerves.

Ultrasound-guided TAP block is a commonly used analgesic technique and is generally used for postoperative analgesia after abdominal surgery^15^. TAP block is performed by administering local anaesthetic medication to the plane between the internal oblique and the transversus abdominis muscle layers and blocking the anterior branch of the T7 to L1 nerves that pass through the plane^9^. The use of TAP block has been found to significantly reduce the VAS scores of patients who have undergone abdominal surgery at 2, 6, 12, and 24 h after surgery, postoperative analgesic requirements, and the incidence of postoperative nausea and vomiting^16^. The time to the first postoperative flatus is also shortened with the use of TAP block^17^. All of these results show that TAP block is an effective and safe technique for relieving pain and facilitating recovery after LC.

A clinical study reported that, compared with posterior TAP block, ultrasound-guided subcostal TAP block more effectively relieves postoperative pain in LC^18^, demonstrating that different techniques of ultrasound-guided TAP block have significant differences in postoperative analgesic effect in LC patients. Compared with oblique subcostal TAP block, ultrasound-guided erector spinae plane block more effectively reduces postoperative pain scores and tramadol consumption in patients after LC^19^, suggesting that subcostal TAP block is not the optimal choice for postoperative analgesia in patients undergoing LC.

The intercostal nerves, which branch out from the spinal cord, run along the intercostal groove and wrap around the front of the ribcage and abdomen and the upper abdominal cutaneous tissue is segmentally innervated by T7–10 intercostal nerves. Pre-emptive ICNB can effectively prevent the stimulation signal caused by surgical trauma from being transmitted to the central nervous system and has the effect of ‘pre-emptive analgesia’^18^. ICNB is commonly used for thoracic anaesthesia and postoperative analgesia^10,20,21^. Fernández Martín *et al*.^22^ reported that ultrasound-guided T6–12 ICNB with levobupivacaine at the midaxillary line provides effective postoperative analgesia in patients undergoing open cholecystectomy.

In the present study, compared with patients undergoing subcostal TAP block, the VAS score of patients undergoing ICNB is significantly reduced from 3 to 8 h after surgery, the mean time to the first use of analgesics after surgery is delayed by approximately 5.5 h, and the median dose of analgesics administered after surgery is reduced by approximately 50%. This shows that ICNB provides a more effective postoperative analgesic effect in LC patients than subcostal TAP block.

Dizziness is common in patients undergoing postoperative analgesia with tramadol^23^ and its incidence is dose-dependent^23,24^. The incidence of postoperative dizziness in patients who undergo transsphenoidal surgery for pituitary adenomas and receive postoperative analgesia with tramadol is 47.5%^25^. In the present study, the incidence of postoperative dizziness in patients with TAP block is 47%, which is consistent with the previous report. However, the incidence of dizziness in patients with ICNB is only 19%, which is significantly lower than that in patients with TAP block. This is probably because the median dose of tramadol for remedial analgesia is significantly lower in the ICNB group than that in the TAP block group (50 (i.q.r. 0–50) *versus* 100 (i.q.r. 0–100) mg respectively). It has been suggested that the incidence of postoperative dizziness is correlated with the analgesic dose of tramadol^26^. Studies have found that tramadol patient-controlled analgesia can effectively alleviate postoperative pain, but can lead to postoperative nausea and vomiting^27^, and that the greater the dose of tramadol for postoperative analgesia, the higher the incidences of postoperative nausea and vomiting^28^. In the present study, the dose of tramadol administered for remedial analgesia is significantly lower in the ICNB group than in the TAP block group which resulted in a higher incidence of postoperative nausea and vomiting in the TAP block group than in the ICNB group.

This study has some limitations. First, the blood concentrations of local anaesthetics after ICNB were not measured. Owing to the abundant blood vessels in the intercostal space and the numerous intercostal nerve blocking sites, the absorption of local anaesthetic into the blood may be faster after ICNB, which may lead to local anaesthetic toxicity. However, none of the patients in the present study experienced local anaesthetic toxicity. This was mainly due to the low concentration of ropivacaine used for ICNB and the total dose of ropivacaine administered being much less than the recommended maximum dose of 200 mg^29^. Second, clinical studies have found that preoperative TAP block can reduce the use of intraoperative anaesthetic analgesics^30^. In this study, no effects of ICNB and TAP block on the demand for intraoperative anaesthetic analgesia were observed, but this will be explored in a subsequent study. Third, the patients in this study underwent LC, which is a relatively short surgical procedure that causes mild surgical trauma. The effect of ICNB on postoperative analgesia in patients with major upper abdominal trauma, such as patients who undergo surgery for stomach and liver diseases, is unclear and needs to be studied in the future. Fourth, it has been found that the performance of multi-point nerve blocks while patients are awake results in patient discomfort and decreased patient acceptance^31,32^. In this study, bilateral ICNB was performed after induction of general anaesthesia; therefore, it did not cause discomfort in patients, which is consistent with previous studies^31^. It is strongly recommended that patients should be given moderate analgesia or sedation to improve patient acceptance when performing a multi-point nerve block^32^. Finally, clinical studies have reported that TAP block does not show significant clinical benefits compared with trocar site infiltration in laparoscopic nephrectomy^33^. In the present study, local anaesthesia was not administered at the trocar site, but this will be included in future studies.

In this study, compared with subcostal TAP block, ultrasound-guided T7–11 ICNB with 0.3% ropivacaine provides better postoperative analgesia with a lower incidence of postoperative dizziness, requires a lower dose of tramadol for remedial analgesia 24 h after surgery, and significantly delays the time of initial use of tramadol for postoperative analgesia.

## Data Availability

The data sets used and/or analysed during the present study are available from the corresponding author upon reasonable request. For ethical reasons, to protect the integrity of the participants, the study data are not publicly available.
